# Clinical Notes

**Published:** 1873-11-15

**Authors:** F. K. Bailey

**Affiliations:** Knoxville, Tennessee


### CLINICAL NOTES.

BY F. K. BAILEY, M.D., KNOXVILLE, TENNESSEE.

### POLYPUS UTERI.

June 13th, 1872, was invited by Dr. S. D. Moses, of this city, to visit with him a patient under his care, a lady, about forty; married, and mother of one child, a son of seventeen. Nervous, lymphatic temperament; nearly bloodless, from loss of blood and suffering.

Last October perceived a growth within the vagina, which increased in size quite rapidly. A few weeks ago, her physicians in a neighboring county attempted to ligate the tumor, but failed in consequence of the wire breaking. About the middle of May last she was brought to this city by her husband, and placed under the care of Dr. Moses.

By reason of weakness, no attempt at removal was made till to-day.

Dr. M. M. Alexander was also invited in. On vaginal exploration with the finger, a large substance was encountered, as large as a placenta, and filling the vagina completely. It came within an inch of the labia, and in form and feel reminded one of a placenta which had descended, and was about to be expelled. On introducing a duck-bill speculum, the tumor could be seen wedged within the vaginal walls, as indicated by digital examination. There was an ichorous discharge, quite offensive, with pus intermixed.

On consultation, it was deemed advisable to attempt to tear away portions of the tumor, in order to test its consistence. Dr. M. succeeded in bringing away three pieces of the size of an English walnut.

Ligation being determined upon, Dr. M. introduced the loop of a ligature made by twisting silver wire and linen cord together. It was very difficult to accomplish this; but after a while it was passed up about to the os uteri, and twisted by means of a pair of forceps. The woman endured the operation very well, and was removed to her bed from the table on which she had been placed.

The tumor appears to be encephaloid in its character, and, with its size, nature, and the previous history of the patient, the prognosis is not favorable.

Thursday, 20th.—Visited the patient with Dr. Moses and Dr. Alexander. Dr. Moses, a few days ago, had tightened upon the ligature, and to day a great portion of the mass was easily removed. There is but little left outside of the ligation, and the remaining portion will doubtless come away soon. General condition of the lady somewhat improved; appetite moderate; less pallor, with rather a brownish hue to the face; pulse 120, but fuller.

September 2d.—Informed by Dr. Moses that Mrs. W. returned to her home in July, but—although the tumor appeared to have been removed—with very bad state of health. Has not heard from her; but the prognosis is exceedingly unfavorable.

December 1st.—I learned, a few days since, that this patient died in October, or November, from exhaustion. The polypus all came away before her death; and it is evident that the operation at an earlier period might have proved successful. The principal points of interest in this case are its rapid growth, probable malignancy, and the large size attained before removal.

I was associated with another physician of this city, a year or two ago, in the removal of a small polypoid growth from the neck of the uterus. The patient was a lady of intelligence and refinement, mother of two or three children, and otherwise in good health, excepting partial deafness. She now is in very good general health. There is reason to believe that in this case a mass of placental dimensions would soon have become developed. Its detection was the result of an ocular examination of the os uteri, with the view to topical treatment for leucorrhœa.

OZAENA.

April 30, 1873.—Mrs. S. M——, about thirty; married; dark complexion; mother of two children, the youngest one year old.

Has had a soreness of the nose for a year or more, attended with the discharge of an offensive purulent nature, and the formation of dark-colored and thick incrustations, which are blown from the nostrils at least once in twenty-four hours. She is still nursing her child, and, until three months since, the menses had been regular in their recurrence. There is nausea at times, with impaired appetite, and a feeling of debility. The pulse is uniformly weak and small. There is a languid, expressionless appearance to the countenance, with depression of spirits. Bowels constipated, and urine scanty. Tongue has thin coat upon the base. Complains of pain in different regions of the lower abdomen, referrable to the uterus or appendages. Directed the use of a weak solution of carbolic acid, as a nasal douche. To take castor oil as laxative.

May 11th.—The nose more troublesome, there being pain and increased soreness; also neuralgia pains in the whole facial region. The incrustations are much lessened in size, and the secretion more healthy. To take, as laxative, the following.

℞.—Fluid ext. sennæ.

Tinct. rhei aa., ℥ i.

Fluid ext. leptandra, ℥ ss.

M. Sig. Teaspoonful at night, and in the morning if necessary, to produce a laxative effect. Continue douche.

15th.—Some improvement since the last report. Bowels open, and appetite improving. Thinks she is pregnant. To continue douche, and the laxative. Is going to the country.

26th.—Came to the city, and I called upon her at the residence of her mother. General appearance improved; countenance more florid; pulse fuller; has some appetite, and is in better spirits. Discharge from the nose lessened, and complains of more acute soreness; every indication that the local affection is healing. To continue douche, and take exercise in the open air, with generous diet.

October 14th.—Have not seen the patient since last report, but I am told she has improved in general health during the summer and fall. Proved to be pregnant, and ere this must have been confined. There is reason to suspect a syphilitic taint in this case, as she has two sisters who have been similarly affected, besides soreness of the throat. A daughter of one of the sisters, October 9, has periosteal ulcerations of both legs, in the anterior aspect, with decided molular swellings. Iod. potass. and other specific remedies have proved beneficial in all the individuals, whenever they can be persuaded to procure and take the medicines.

We encounter very often such cases here; and there are multitudes who linger on through a wretched life, bearing in their bodies the traces, in one stage or another, of an old syphilitic taint. Climatic influences modify more or less the character of such affections.

I have now in mind the case of a man about forty-five, who came here from Virginia, in 1870. Sloughing and exfoliation had become so extensive that the stench was intolerable. A syphilitic affection of twenty years' standing was said to be the cause of this terrible affection. Still, this man was in active business in both States, and was able to be about most of the time. A portion of the nasal bones have been removed within three years, but with what result I am not advised.

